# Gas-Sensing Property of TM-MoTe_2_ Monolayer towards SO_2_, SOF_2_, and HF Gases

**DOI:** 10.3390/molecules27103176

**Published:** 2022-05-16

**Authors:** Aijuan Zhang, Qunfeng Dong, Yingang Gui, Jinfang Li, Feng Wan

**Affiliations:** 1 College of Physics and Electronic Engineering, Xianyang Normal University, Xianyang 712000, China; zhangaijuan2019@163.com (A.Z.); qunfengdong1028@163.com (Q.D.); lijinfang928@163.com (J.L.); wanfeng1101@163.com (F.W.); 2 College of Engineering and Technology, Southwest University, Chongqing 400715, China

**Keywords:** MoTe_2_ monolayer, SO_2_, SOF_2_, HF, DFT

## Abstract

Detecting the characteristic decomposition products (SO_2_, SOF_2_, and HF) of SF_6_ is an effective way to diagnose the electric discharge in SF_6_-insulated equipment. Based on first-principles calculations, Au, Ag, and Cu were chosen as the surface modification transition metal to improve the adsorption and gas-sensing properties of MoTe_2_ monolayer towards SO_2_, SOF_2_, and HF gases. The results show that Au, Ag, and Cu atoms tend to be trapped by T_H_ sites on the MoTe_2_ monolayer, and the binding strength increases in the order of Ag < Au < Cu. In gas adsorption, the moderate adsorption energy provides the basis that the TM-MoTe_2_ monolayer can be used as gas-sensing material for SO_2_, SOF_2_, and HF. The conductivity of the adsorption system changes significantly. The conductivity decreases upon gases adsorption on TM-MoTe_2_ monolayer, except the conductivity of Ag-MoTe_2_ monolayer increases after interacting with SOF_2_ gas.

## 1. Introduction

In the compact design of high-voltage equipment and gas insulation systems in the power industry [1], SF_6_ gas has been widely used as an insulation medium due to its comprehensive advantages, such as high dielectric strength, strong electronegativity, thermal stability, chemical inertness, and non-toxicity [2,3]. However, SF_6_ gas inevitably decomposes to toxic and corrosive by-products under electric discharge [4]. Corona, spark, and arc discharge are three typical types of electric discharge observed in SF_6_-insulated equipment [5]. Under the electric discharge, the low-sulfur fluorides produced by ionizing SF_6_ gas will quickly react with trace moisture and impurities in the gas insulation system, forming some common stable decomposition products, including SO_2_, SOF_2_, and HF [6,7,8]. If these decomposition products are not handled properly, they will reduce the insulation strength of the filling gas, and be harmful to the environment and human health [9]. In addition, these acid gases will also corrode the original insulation device inside the gas insulation system, aggravating electric discharge and further affecting the safe and stable operation of the power system [10]. Online detection of the concentration of SO_2_, SOF_2_, and HF gases in the gas insulation system is crucial to ensure the safe running of equipment [11]. Therefore, it is urgent to explore suitable gas-sensitive materials for high-efficiency detection of the SF_6_ decomposed gases.

Based on the catalytic performance and unique electrical structure, two-dimensional layered materials-based chemical sensors have been extensively explored and studied in broad application prospects, such as equipment testing, environmental diagnosis, and industrial manufacturing [12,13]. Graphene-like materials, such as transition metal dihalides (TMD), InN monolayers, hexagonal boron nitride (h-BN), and carbon nitride compounds, have a large specific surface area, and are resistant to strong acids and alkalis, and high temperatures [14,15,16]. Its adsorption and gas-sensitivity properties to gases have been extensively studied [17,18]. Compared with other TMD, MoTe_2_ has lower binding energy and a larger bond length [19,20]. TMD-based gas sensors have attracted broad focus in recent years [21]. Wang et al. studied the gas-sensing potential of MoTe_2_ monolayer to SF_6_ decomposition products based on theoretical calculations [22]. Feng et al. developed a MoTe_2_-based gas sensor for NH_3_ and NO_2_ detection, with an excellent recovery rate and high sensitivity [23]. These previous studies have significantly enhanced the chemical interaction with specific gases, providing a promising candidate for SF_6_ decomposition product detection.

The introduction of transition metal atom modification on the surface of two-dimensional layered materials effectively improves surface activity and gas-sensing performance [24,25]. In particular, nano-noble metals, such as Au, Ag, and Cu, may show better surface performance [26]. This is because transition metal atom modification increase the chemical activity and electron mobility of pristine materials, opening up a new perspective on exploring high-performance gas sensor [27]. Based on density functional theory (DFT) calculations, transition metals (Au, Ag, and Cu) were selected as modifying atoms on the MoTe_2_ surface in this study. Then, SO_2_, SOF_2_, and HF gas molecules are adsorbed on three transition metals (Au, Ag, and Cu) modified MoTe_2_ monolayer, abbreviated as TM-MoTe_2_. The surface modification and gas-sensing mechanism have been studied by analyzing the geometric structures and electronic properties.

## 2. Computational Details

All calculations are performed based on DFT calculations [28]. The Perdew–Burke–Ernzerhof (PBE) function of the generalized gradient approximation method (GGA) was selected to approximate the exchange and correlation of electrons [29]. DFT Semi-core Pseudopot (DSPP) was selected to eliminate the relativistic effect of TM atoms in the core processing [30]. The p-orbital dual-value plus polarization function (DNP) was used as the atomic orbital basis set, which increases the calculation accuracy of hydrogen bonds [31]. The Grimme method performed DFT-D correction on Ag and Cu modified MoTe_2_ models. While DFT-D2 method by TS was used to analyze the intermolecular forces and long-range interactions in the Au-modified MoTe_2_ model.

A 7 × 7 × 1 Monkhorst pack grid was wet for Brillouin sampling. A static calculation with a self-consistent field convergence accuracy of 10^−6^ Ha, a global orbit cut-off radius of 4.9 Å, and a smearing of 0.005 Ha were used to ensure the smooth convergence of the entire system [32]. According to the principle of periodic boundary conditions to eliminate the boundary effect, a single layer of MoTe_2_ was constructed in a 4 × 4 × 1 supercell containing 16 Mo atoms and 32 Te atoms. A vacuum interval of 20 Å is used to avoid mutual influence between adjacent layers [33]. Four possible modification sites of TM atoms on MoTe_2_ were considered, including T_H_, T_Mo_, T_Te_, and T_B_. T_H_ is the location above the center of the MoTe_2_ hexagonal ring, T_Mo_ is the location on the top of the Mo atom, T_Te_ is the location on the top of the Te atom, and T_B_ means the location on the bridge between two Te atoms. The binding energy (*E*_b_) of TM atoms on the MoTe_2_ monolayer is defined in Equation (1). Where *E*_TM-MoTe2_, *E*_TM_, and *E*_MOTe2_ represent the energy of TM-MoTe_2_ monolayer, TM atom, and pristine MoTe_2_ monolayer, respectively.

*E_b_* = *E*_TM-MoTe2_ − *E*_TM_ − *E*_MoTe2_ (1)

After obtaining the most stable TM-MoTe_2_ monolayer structure, various possible adsorption positions of the gas molecule on TM-MoTe_2_ were considered to study the adsorption behavior. Gas molecules undergo significant displacement and move to the highest stability position after structural optimization. The most stable configuration for gas molecule adsorption is determined by the largest adsorption energy (*E*_ads_) calculated as Equation (2):

*E*_ads_ = *E*_TM-MoTe2/gas_ − *E*_TM-MoTe2_ − *E*_gas_ (2)

where *E*_TM-MoTe2/gas_ is the total energy of the adsorption system, while *E*_TM-MoTe2_ and *E*_gas_ are the total energy of separated TM-MoTe_2_ monolayer and gas molecules, respectively. The Mulliken atomic charges method is used to analyze the charge transfer. A negative value of charge transfer indicates the electrons transfer from TM-MoTe_2_ monolayer to gas molecules.

## 3. Results and Discussion

### 3.1. Structural Optimization of Gas Molecules and TM-MoTe_2_ Monolayer

**Figure 1:**
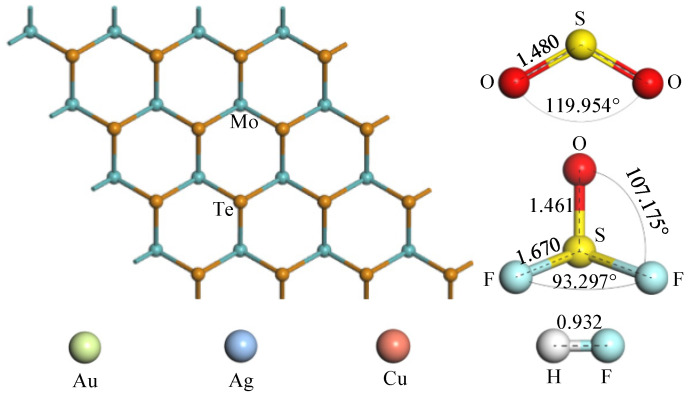
The optimized structures of the pristine MoTe_2_, TM atoms, SO_2_, SOF_2_, and HF gases. The unit is Å.

As shown in Figure 1, the pristine MoTe_2_ monolayer, TM atoms, SO_2_, SOF_2_, and HF gas molecules were optimized. The pristine MoTe_2_ monolayer structure is composed of a hexagonal pattern with Se atoms and Mo atoms intersecting. The Mo-Te bond length of the MoTe_2_ monolayer structure before TM modification is 2.758 Å. SO_2_ gas molecule has a broken-line spatial configuration, which structure keeps good agreement with the reported theoretical and experimental results. The central S atom and the other two O atoms are bonded by σ bonds, and the O-S-O structure forms an angle of 119.954° and an S-O bond length of 1.480 Å. SOF_2_ gas molecules belong to a three-dimensional structure due to the multiple-valence property. The bond lengths of the S-F bond and the S-O bond are 1.670 Å and 1.461 Å, respectively, and the angles of the O-S-F structure and the F-S-F structure are 107.175° and 93.297°, respectively. HF gas is in a linear structure with a bond length of 0.932 Å.

**Figure 2:**
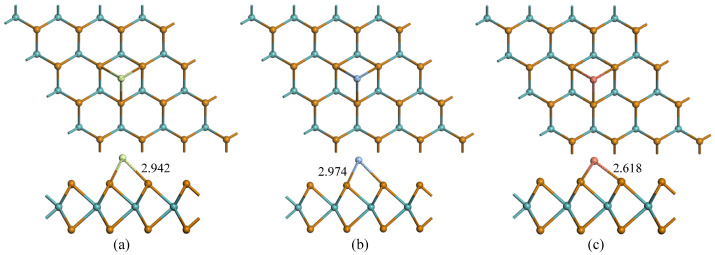
Top view and side view of: (**a**) Au-MoTe_2_; (**b**) Ag-MoTe_2_; (**c**) Cu-MoTe_2_. The unit is Å.

First, the geometric structure and electronic properties of the TM-MoTe_2_ monolayer were studied. Liu et al. achieved geometric relaxation of TM modification on the pristine MoTe_2_ monolayer through four possible positions [34]. After complete optimization, it can be seen that the TM atoms prefer to be trapped at the TH site (located above the center of the MoTe_2_ hexagonal ring). Therefore, this paper has three different TM atoms in the TH position for geometric optimization. It is worth noting that the three TM atoms form three bonds with three adjacent Te atoms after optimization, resulting in a certain degree of structural distortion in each system. However, in the optimized configuration, the lengths of the three TM-Te bonds are equal. It can be seen from Figure 2 that the average length of the TM-Mo bond in the TM-MoTe_2_ model is 2.942 Å, 2.974 Å, and 2.618 Å, respectively.

**Figure 3:**
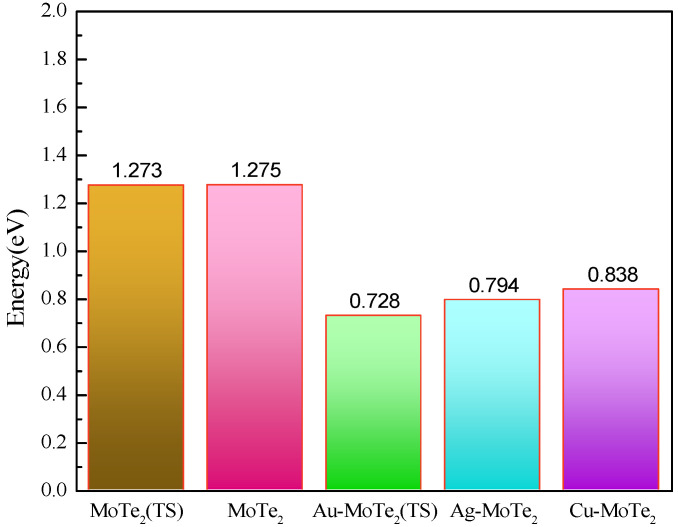
The bandgaps of the pristine MoTe_2_ monolayer and TM-MoTe_2_ monolayer.

Table 1 shows that the bonding strengths of TM atoms on MoTe_2_, which increase in the order of Ag < Au < Cu, and the binding energies are 1.61 eV, 1.47 eV, and 1.13 eV, respectively. It shows that Au, Ag, and Cu of the same group can interact with the adjacent undercoordinated Mo atoms. The electron transfer from the TM atoms to the MoTe_2_ monolayer is −0.238 e, 0.007 e, and −0.104 e, respectively, which is crucial to the chemical activity and sensitivity of the TM modified MoTe_2_ monolayer. As shown in Figure 3, the band gaps of the MoTe_2_ single layer under TS optimization and Grimme optimization are 1.273 eV and 1.275 eV, respectively, which is not much different overall. All of the bandgaps of the TM-MoTe_2_ monolayer reduce, especially the bandgap of Au-MoTe_2_ reduces to 0.728 eV. The band gaps of Ag-MoTe_2_ and Cu-MoTe_2_ are also respectively reduced to 0.794 eV and 0.838 eV, which is very important for the performance of the activated material.

### 3.2. Adsorption of Gas Molecules on TM-MoTe_2_ Monolayer

**Figure 4:**
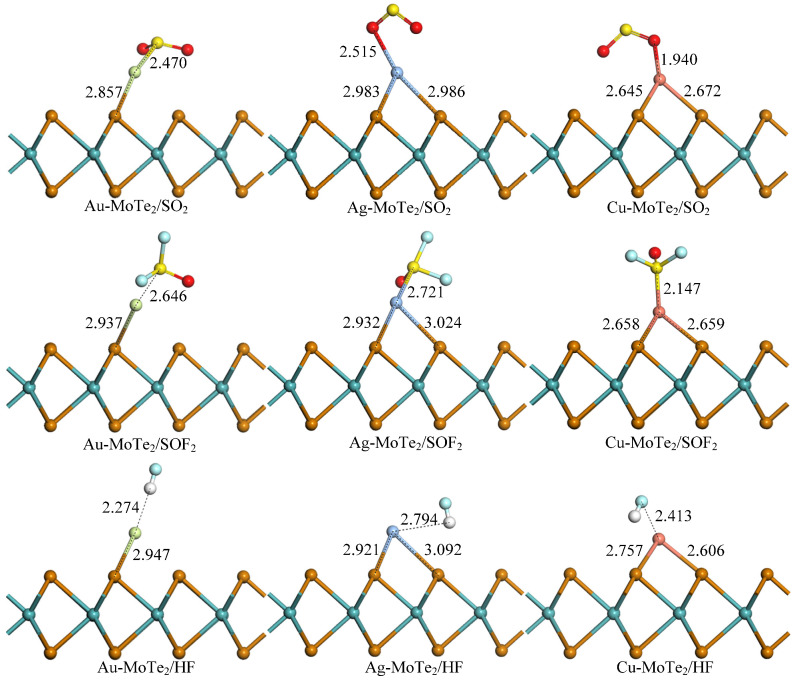
Adsorption systems of the gas molecule on TM-MoTe_2_ monolayer. The unit is Å.

The adsorption structures of SO_2_, SOF_2_, and HF gas molecules on the most stable TM-MoTe_2_ monolayer structure were obtained, as shown in Figure 4. Table 2 lists the adsorption energy and charge transfer between the TM-MoTe_2_ adsorption system and SO_2_, SOF_2_, and HF gas molecules. The calculated *E*_ads_ ranges from −0.23 eV to −1.18 eV, determining whether gas adsorption processes belong to chemical or physical adsorption. *Q*_T_ ranges from +0.031 e to −0.341 e, indicating the redistribution of the electrons for all systems. It can be seen that SOF_2_ prefers to interact with the TM-MoTe_2_ monolayer by the S atom of SO_2_F_2_ approaching the TM atom. This is because the unique molecular configuration of SOF_2_ makes the S atom more multivalent.

It can be seen from Figure 4 that the structure of the TM-MoTe_2_ monolayer has undergone significant deformation after the adsorption of SO_2_, SOF_2_, and HF gas molecules. In particular, the Au atom undergoes a significant movement from the initial modification position, the position above the center of the MoTe_2_ hexagonal ring to a position partial to the top of the Te atom, and two of the original three Au-Te bonds are broken. The length of the remained Au-Te bond changes to 2.857 Å, 2.937 Å, and 2.947 Å when SO_2_, SOF_2_, and HF gases adsorb on the Au-MoTe_2_ monolayer, respectively. On the other hand, the Ag and Cu modified MoTe_2_ monolayer have not undergone significant geometric deformation during the adsorption of SO_2_, SOF_2_, and HF gases, though the Ag-Te bond and Cu-Te bond are elongated to some extent. It can be determined that the Ag-MoTe_2_ adsorption system is similar to the Cu-MoTe_2_ adsorption system. The O atoms in the SO_2_ gas molecule and the S atoms in the SOF_2_ gas molecule are stably captured by the Ag atom and Cu atom by forming corresponding chemical bonds: Ag-O (2.515 Å), Ag-S (2.721 Å), Cu-O (1.940 Å), and Cu-S (2.147 Å).

### 3.3. TDOS and PDOS Distribution of Gas Adsorbed TM-MoTe_2_ Monolayer

**Figure 5:**
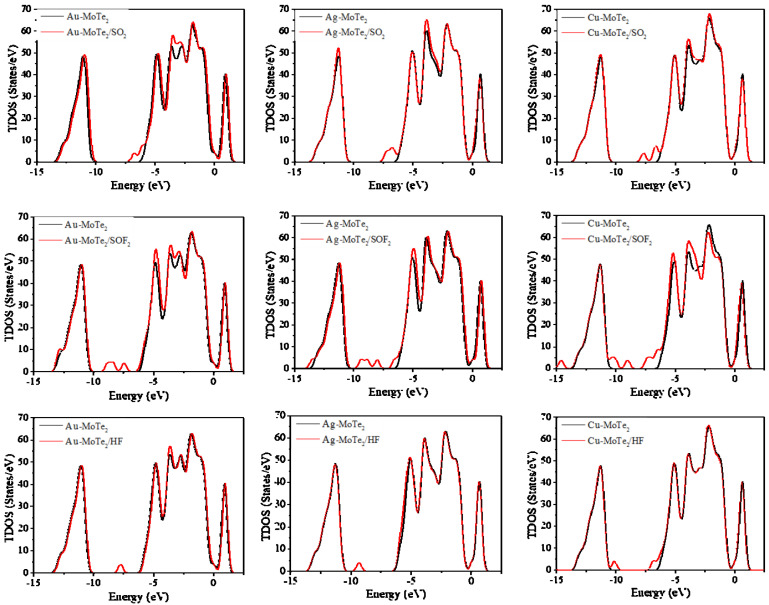
TDOS distribution of SO_2_, SOF_2_, and HF adsorbed TM-MoTe_2_ systems.

Figure 5 shows the TDOS distribution of TM-MoTe_2_ monolayers before and after SO_2_, SOF_2_, and HF gas adsorption. It can be seen that the TDOS distribution of the Au-MoTe_2_ monolayer shifts to the right as a whole after the adsorption of SO_2_, SOF_2_, and HF gases, proving that the modification of Au atoms improves the chemical activity and conductivity of the adsorption system. In addition, due to the activated state of the adsorbed SOF_2_ gas molecule, the TDOS distribution of the SOF_2_ adsorbed TM-MoTe_2_ system showed a continuous new peak between −10 eV and −5 eV. Moreover, the TDOS distribution of the SOF_2_ gas adsorption system fluctuates the most in all distributions due to a certain amount of charge transfer during the adsorption process. On the right side of the Fermi level, the TDOS distribution decreases after SO_2_ gas adsorption on the Ag-MoTe_2_ monolayer and Cu-MoTe_2_ monolayer, indicating that the filled electrons reduce, and a strong chemical effect occurs during the adsorption process. The TDOS distribution of the HF adsorbed Au-MoTe2 system shifts slightly to the right, but the TDOS distribution of the other two HF gas adsorption systems nearly does not change.

**Figure 6:**
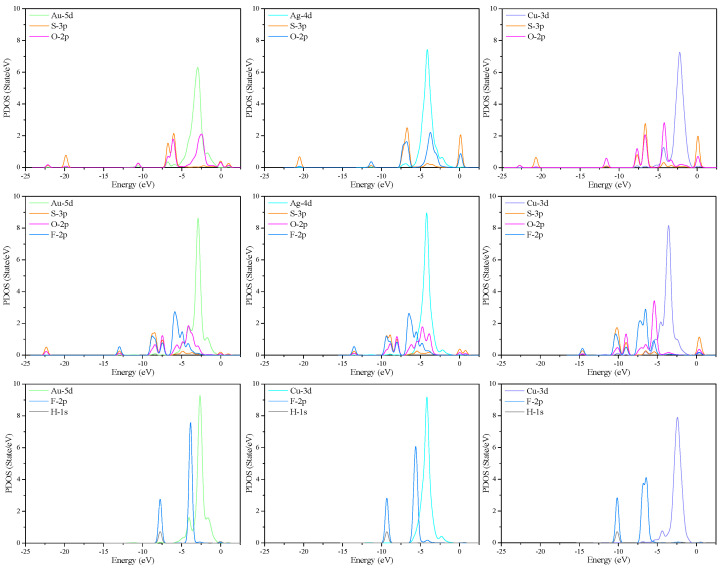
PDOS distribution of SO_2_, SOF_2_, and HF adsorbed TM-MoTe_2_ systems.

PDOS analysis was performed to understand the electronic behavior of TM-MoTe_2_ monolayer when adsorbing SO_2_, SOF_2_, and HF gases, as shown in Figure 6. It can be found that the 5*d*, 4*d* and 3*d* orbits of Au, Ag, and Cu atoms have a significant influence on their respective TDOS distributions due to their mental activities. It can be seen from the PDOS distribution of each adsorption system that the *d* orbits of TM atoms highly hybridize with the *p* orbits of S atoms or O atoms of the adsorbed SO_2_ gas molecules and SOF_2_ gas molecules. It confirms the stable formation of TM-S or TM-O bonds in the TM-MoTe_2_ adsorption system. In the TDOS distribution of all TM-MoTe_2_/HF adsorption systems, a slight fluctuation between −12.5 eV and −7.5 eV appears as a new peak derived from the 2*p* and 1*s* orbits of HF gas molecule. These orbital interactions indicate that SO_2_, SOF_2_, and HF gases have an ideal adsorption effect on the TM-MoTe_2_ monolayer, which leads to the redistribution of electrons on the substrate and a change of conductivity to a large extent.

### 3.4. Molecular Orbital Analysis of Gas Adsorbed TM-MoTe_2_ Monolayer

**Figure 7:**
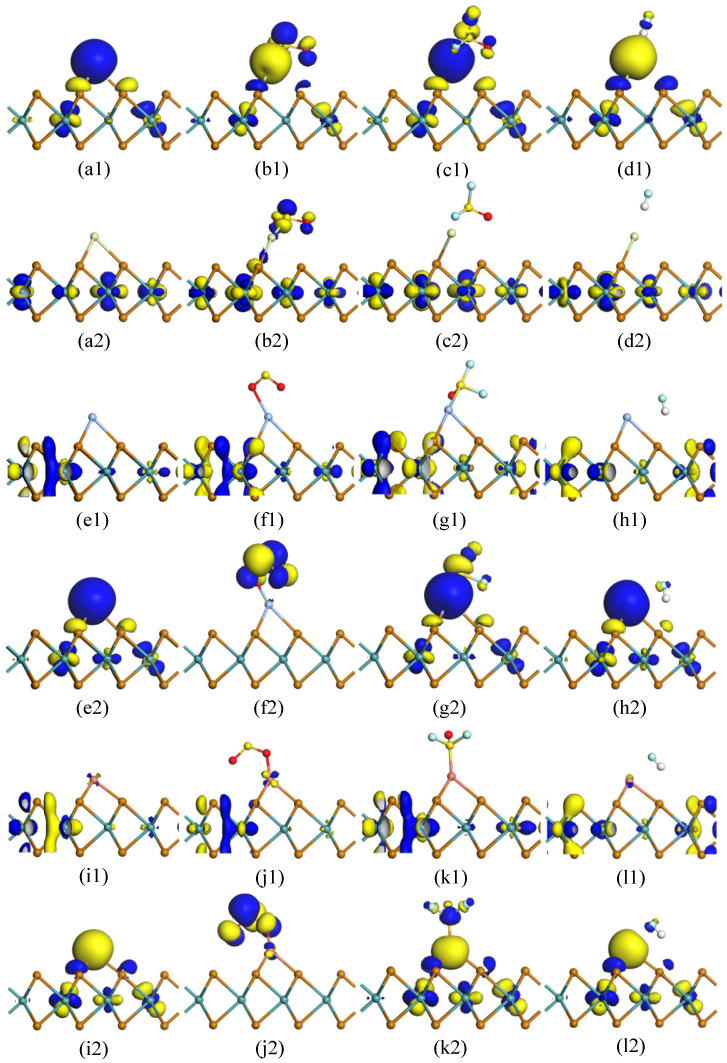
HOMO and LUMO distribution of TM-MoTe_2_ monolayer before and after gas adsorption. (**a1**) Au-MoTe_2_-HOMO, (**a2**) Au-MoTe_2_-LUMO, (**b1**) Au-MoTe_2_/SO_2_-HOMO, (**b2**) Au-MoTe_2_/SO_2_-LUMO, (**c1**) Au-MoTe_2_/SOF_2_-HOMO, (**c2**) Au-MoTe_2_/SOF_2_-LUMO, (**d1**) Au-MoTe_2_/HF-HOMO, (**d2**) Au-MoTe_2_/HF-LUMO, (**e1**) Ag-MoTe_2_-HOMO, (**e2**) Ag-MoTe_2_-LUMO, (**f1**) Ag-MoTe_2_/SO_2_-HOMO, (**f2**) Ag-MoTe_2_/SO_2_-LUMO, (**g1**) Ag-MoTe_2_/SOF_2_-HOMO, (**g2**) Ag-MoTe_2_/SOF_2_-LUMO, (**h1**) Ag-MoTe_2_/HF-HOMO, (**h2**) Ag-MoTe_2_/HF-LUMO, (**i1**) Cu-MoTe_2_-HOMO, (**i2**) Cu-MoTe_2_-LUMO, (**j1**) Cu-MoTe_2_/SO_2_-HOMO, (**j2**) Cu-MoTe_2_/SO_2_-LUMO, (**k1**) Cu-MoTe_2_/SOF_2_-HOMO, (**k2**) Cu-MoTe_2_/SOF_2_-LUMO, (**l1**) Cu-MoTe_2_/HF-HOMO, (**l2**) Cu-MoTe_2_/HF-LUMO.

The adsorption of SO_2_, SOF_2_, and HF gases on the TM-MoTe_2_ monolayer was analyzed by molecular orbital analysis. According to the molecular orbital theory, the highest occupied molecular orbit (HOMO) and lowest unoccupied molecular orbit (LUMO) distributions of the adsorption system were calculated as shown in Figure 7. The yellow and blue areas in Figure 7 represent the positive and negative phases of the wave function. Table 3 shows the energy gap (*E*_g_) between HOMO and LUMO, which helps evaluate the change in conductivity.

Molecular orbit is an effective method to evaluate the probability of electron transfer between characteristic molecules and the surface of TM-MoTe_2_. This confirms the previous DOS analysis that SO_2_, SOF_2_, and HF gas molecules are non-magnetic molecules during adsorption. At the same time, the *E*_g_ of the TM-MoTe_2_ monolayer changes after the gas molecules’ adsorption. For the Ag-MoTe_2_ system, the *E*_g_ of the adsorption system decreases after SOF_2_ gas molecules adsorption. While for other TM-MoTe_2_ systems, the *E*_g_ of the adsorption system increases after the gas adsorption. As the LUMO distribution obviously increases around the gas adsorption site, and HOMO distribution on Ag-MoTe_2_ slightly increases when SOF_2_ adsorption on the Ag-MoTe_2_ system. As a result, the electron transition from HOMO to LUMO becomes easier, and *E*_g_ decreases simultaneously. Therefore, the conductivity of the Ag-MoTe_2_ monolayer increases after interacting with SOF_2_ gas, while the conductivity of other adsorption systems decreases. As shown in Figure 7, the adsorption of gas molecules causes the redistribution of electrons in TM-MoTe_2_, which changes the energy of HOMO and LUMO accordingly, which matches the changing trend of *E*_g_.

## 4. Conclusions

Based on first-principles calculations, this paper studies the stable structure of TM (Au, Ag, and Cu) modification on the MoTe_2_ monolayer. Then the adsorption structures of the characteristic decomposition products of SF_6_ (SO_2_, SOF_2_, and HF) on the TM-MoTe_2_ monolayer were calculated. By analyzing the adsorption structure, adsorption energy, charge transfer, adsorption distance, TDOS, PDOS, and molecular orbit, the adsorption performance and electronic behavior of TM-MoTe_2_ monolayer towards SO_2_, SOF_2_, and HF gases were explored. TM atoms tend to be trapped by T_H_ sites on the MoTe_2_ monolayer with a binding strength of Ag <Au < Cu. The adsorption energy of the TM-MoTe_2_ monolayer to SO_2_, SOF_2_, and HF gas is moderate, indicating that they are suitable gas-sensing materials for detecting SO_2_, SOF_2_, and HF gases. The adsorption of SO_2_, SOF_2_, and HF gases on the TM-MoTe_2_ monolayer leads to the redistribution of electrons in the TM-MoTe_2_ systems, affecting its conductivity to a large extent. After SOF_2_ adsorption on the Ag-MoTe_2_ monolayer, the conductivity increases along with the decreased *E*_g_, while the other gas adsorption on the TM-MoTe_2_ monolayer leads to an increase of *E*_g_ and a decrease in conductivity. Based on the different change rules of conductivity of the systems, it realizes the selective detection of the mixed decomposition products.

## Figures and Tables

**Table 1:** Binding energy and charge transfer of TM atoms modification on MoTe_2_.

Modification Site	*E*_bind_ (eV)	*Q*_T_ (e)
Au-MoTe_2_	−1.47	−0.238
Ag-MoTe_2_	−1.13	0.007
Cu-MoTe_2_	−1.61	−0.104

**Table 2:** Adsorption energy and electron transfer of gas molecules on TM-MoTe_2_ monolayer.

Parameters	*E*_ads_ (eV)	*Q*_T_ (e)
Au-MoTe_2_/SO_2_	−0.98	−0.259
Ag-MoTe_2_/SO_2_	−0.81	−0.341
Cu-MoTe_2_/SO_2_	−1.18	−0.316
Au-MoTe_2_/SOF_2_	−0.49	−0.147
Ag-MoTe_2_/SOF_2_	−0.4	−0.158
Cu-MoTe_2_/SOF_2_	−0.6	0.077
Au-MoTe_2_/HF	−0.23	−0.033
Ag-MoTe_2_/HF	−0.32	−0.007
Cu-MoTe_2_/HF	−0.33	0.031

**Table 3:** Molecular orbits and energy gaps of before and after gas adsorption on TM-MoTe_2_.

Adsorption Structure	*E*_HOMO_ (eV)	*E*_LUMO_ (eV)	*E*_g_ (eV)
Au-MoTe_2_	−4.599	−3.646	0.953
Au-MoTe_2_/SO_2_	−4.898	−3.837	1.061
Au-MoTe_2_/SOF_2_	−4.844	−3.81	1.034
Au-MoTe_2_/HF	−4.926	−3.891	1.035
Ag-MoTe_2_	−5.143	−4.163	0.98
Ag-MoTe_2_/SO_2_	−5.333	−4.218	1.115
Ag-MoTe_2_/SOF_2_	−5.306	−4.435	0.871
Ag-MoTe_2_/HF	−5.225	−4.191	1.034
Cu-MoTe_2_	−5.17	−4.136	1.034
Cu-MoTe_2_/SO_2_	−5.306	−4.218	1.088
Cu-MoTe_2_/SOF_2_	−5.333	−4.136	1.197
Cu-MoTe_2_/HF	−5.197	−4.082	1.115

## Data Availability

Not applicable.

## Sample Availability

Samples of the compounds are not available from the authors.
